# Towards Meaningful Consumer and Community Involvement in Health Research: A Qualitative Study of Consumer and Researcher Experiences

**DOI:** 10.1111/hex.70620

**Published:** 2026-03-01

**Authors:** Kimberley A. Baxter, Jennifer Muller, David A. Copland, Nadine E. Foster, Emmah Doig, Jessica A. Schults, Karina R. Charles, Adrienne Young, Silvia Manzanero, Tanya Smyth, Lisa Anemaat

**Affiliations:** ^1^ STARS Education and Research Alliance, Surgical Treatment and Rehabilitation Service (STARS) The University of Queensland and Metro North Health Brisbane Australia Australia; ^2^ School of Health and Rehabilitation Sciences, Faculty of Health, Medicine and Behavioural Sciences The University of Queensland Brisbane Australia; ^3^ Queensland Aphasia Research Centre The University of Queensland Brisbane Australia; ^4^ School of Nursing, Midwifery and Social Work, Faculty of Health, Medicine and Behavioural Sciences The University of Queensland Brisbane Australia; ^5^ Herston Infectious Diseases Institute (HeIDI) Metro North Health Brisbane Australia; ^6^ Dietetics and Food Services, Royal Brisbane and Women's Hospital Brisbane Australia; ^7^ Centre for Health Services Research The University of Queensland Brisbane Australia; ^8^ Jamieson Trauma Institute (JTI) Metro North Health Brisbane Australia; ^9^ School of Medicine Queensland University of Technology Brisbane Australia

**Keywords:** community participation, consumer and community involvement, consumer engagement, health research, interview, lived experience, patient and public Involvement

## Abstract

**Background:**

Consumer and community involvement (CCI) in health research is recognised as an essential component of ethical, effective and relevant research practice. While the recommended principles that enable high‐quality CCI are well established, consistent implementation remains a challenge.

**Objective:**

To inform consumer involvement strategies and practices, this study explored the experiences of researchers and consumers involved in CCI in research at a large metropolitan health service precinct in Brisbane, Australia.

**Methods:**

Semi‐structured interviews were used to explore participants' experiences of CCI. Researchers and consumers who had undertaken CCI activities were purposively sampled from two hospitals and six research institutes. Thematic analysis using the framework method was employed to interpret the data. Coding was conducted both inductively and deductively, utilising a data‐driven approach guided by the study's objectives.

**Results:**

A total of 27 participants contributed (researchers, *n* = 17; consumers, *n* = 10). Five themes captured supportive and challenging elements of CCI and participants' visions for meaningful CCI in research. Themes included ‘Laying the Groundwork’, which reflects the foundational elements needed for meaningful partnering, and ‘Navigating the Human Factor’, which captures the interpersonal dynamics that shape CCI. ‘Bridging Methods’ described methodological approaches to CCI. Participants' challenging experiences with organisational structures and culture were represented by ‘Opportunities: Organisational Barriers’, while ‘Paving the Way Forward’ pointed to practical strategies for embedding CCI meaningfully and sustainably into research processes.

**Conclusion:**

This study shows that while CCI in health research is highly valued, it faces challenges like organisational barriers, administrative load and limited support. Meaningful CCI depends on early relationship building, role clarity, flexible processes and culturally safe, trauma‐informed practices. To turn policy into action, research‐active health services must streamline systems and create structures for long‐term, inclusive involvement. Researcher training should cover CCI principles, as well as interpersonal and facilitation skills crucial for building successful and meaningful involvement partnerships.

**Patient or Public Contribution:**

This study benefited from the involvement of a health consumer partner (J.M.) throughout all stages of the research. Our consumer partner contributed to (1) drafting the initial grant proposal; (2) developing the research protocol, methods and processes; (3) research governance meetings; (4) the design and refinement of interview guides to ensure relevance and clarity of questions; (5) providing feedback in the form of sense checking developing themes to refine interpretation of findings; and (6) critically reviewing and providing feedback on manuscript drafts.

## 1 Introduction

Consumer and community involvement (CCI), also known as patient and public involvement (PPI), is widely recognised as a critical component of high‐quality, relevant and ethically sound health research [1]. While terminology differs internationally, the term CCI is used in Australia. CCI in research refers to the active partnership in the design, conduct and dissemination of research of individuals with lived experience of health services, including patients, their families and the community or public who interact with the healthcare system [2]. Unlike public engagement activities or participation as a research subject, CCI in research entails active roles within research teams, such as advisory positions, contributing to decision‐making, shaping research priorities and co‐producing outputs. CCI has been shown to enhance the relevance, quality and impact of research across the research cycle [3], increase public trust and reduce research waste [4, 5]. CCI in health research has been associated with a range of positive impacts, including improved study design, increased relevance to end‐users, and enhanced recruitment and retention of participants [6]. There is also an ethical imperative to involve those directly impacted by health research in shaping the research agenda and process [7].

Despite widespread endorsement of CCI by funding bodies, health organisations and policy‐makers [8, 9], meaningful and collaborative CCI in health research remains inconsistently implemented [2]. Tokenistic involvement persists, with consumers included but lacking real influence on research prioritisation and decision‐making [10, 11]. There is also a lack of system processes and structures in place to support researchers and consumers in meaningful and active CCI [12, 13, 14, 15]. Guiding principles and values for effective CCI in health research are well articulated by funding bodies and frameworks. The Australian National Health and Medical Research Council (NHMRC), together with the national peak body for consumers in Australia (Consumer Health Forum), has developed a statement that outlines the roles, responsibilities and ethical foundations of CCI, to guide research institutions, researchers and consumers in active involvement [9]. Internationally, similar principles are championed by the National Institute for Health Care Research (NIHR) [16], the Canadian Institutes of Health Research [17] and the Patient‐Centred Outcomes Research Institute in the United States [18]. Together, these guidelines support the growing consensus that CCI is both a methodological strength and an essential component of high‐quality health research.

There is a disparity between consumers' expressed needs and the ways health researchers design and integrate CCI in research, creating a barrier to the implementation of best practice CCI principles [19]. There is also a recognition of the importance of contextual factors and local settings for the successful implementation of CCI [20]. Qualitative enquiry offers valuable insights into relational, organisational and systemic factors that influence practices and help address complex questions [21]. These insights are particularly important for informing local strategies that aim to strengthen consumer–researcher partnerships and embed CCI more meaningfully into routine research practices. By examining the experiences of consumers and researchers, CCI implementation in health research can enable more meaningful involvement. To inform CCI strategies and practices, this study aimed to explore the experiences of consumers and researchers involved in CCI in research at a large metropolitan health service precinct in Brisbane, Australia.

## 2 Methods

### 2.1 Study Design

This study was part of a broader research programme that included an online survey, interviews and a collaborative priority‐setting workshop [22]. This paper presents the results of a qualitative thematic analysis of the semi‐structured interviews. The reporting was guided by the Consolidated Criteria for Reporting Qualitative Research (COREQ) [23] and Guidance for Reporting Involvement of Patients and the Public (GRIPP2) [24]. The GRIPP2 checklist is provided in Supporting Information S1: 1 for transparency of CCI practice. The results of the online survey and collaborative priority‐setting workshop will be reported elsewhere.

### 2.2 Setting

The study took place across a large metropolitan health service precinct in Brisbane, Queensland, which includes two hospitals and numerous research centres conducting preclinical, clinical and health services research. Participants were invited from two research‐active public hospitals and six research centres co‐located within the health service.

### 2.3 Participant Recruitment and Sampling

Participants included researchers, clinician‐researchers and university academics, collectively referred to as ‘researchers’, and consumers with experience of consumer involvement in a research project, process or initiative. Recruitment strategies included staff newsletters, hospital notices, research networks, email lists and consumer networks, with snowball methods used to distribute study information. Participants who had consented and completed a survey were then able to express interest in being contacted to participate in a semi‐structured interview. Participants were contacted up to three times to confirm their willingness to participate in the study. All participants provided informed consent before their participation.

### 2.4 Data Collection

Two semi‐structured interview guides were created for researcher and consumer participants, which were informed by CCI in research policy and guidelines in the Australian context [9, 13, 25] (see Supporting Information S1: 2). The guides included open‐ended questions about participants' experiences with CCI in research, including perceived challenges and opportunities for improvement. Our consumer collaborator (J.M.) contributed to the development and reviewed the interview question guides. Minor adjustments were made to the interview guides for improved readability and context, based on the feedback received. A pilot test of the researcher interview guide was carried out on November 18, 2024, with a colleague experienced in involving consumers in research, who was not part of the research team. No modifications were made to the interview guide following the pilot, and the data did not contribute to the study.

Interviews were conducted by the first author between November 20, 2024 and February 10, 2025, either in person at the health service or via Microsoft Teams [26], depending on the participant's preference. Interviews were audio‐recorded and transcribed using the transcription features in Microsoft Teams or Word. The interviewer took field notes after the interviews, relating to the participant's background, such as their lived experience or research topics. Generated transcripts were manually checked and corrected by the first author for accuracy against the audio recording. Member checking was incorporated during the interview by paraphrasing and summarising participants' contributions for validation. Transcripts were not returned to participants for checking and were de‐identified before coding and analysis. The sample target planned was 16 (*n* = 8 researchers and *n* = 8 consumers). The final sample size was influenced by the number of expressions of interest from participants and the adequacy of data collection to address the research aims. Demographic characteristics were collected via the online survey to describe the interview sample.

### 2.5 Data Processing and Analysis

Thematic analysis using the Framework Method was employed to interpret the data and identify themes and relationships [27]. The Framework Method is a systematic and flexible approach to analysing qualitative data, commonly used in healthcare research [28]. It was chosen for its interconnected approach to analysis. Descriptive or conceptual labels (codes) were applied to excerpts of raw data. The coding approach combined inductive and deductive coding, informed by the study's aims, along with data‐driven codes. Two researchers (K.A.B. and L.A.) independently coded two transcripts and discussed the differences and similarities in the coding applied. Based on these initial codes, a preliminary coding framework was developed, including codes and sub‐codes. Two additional transcripts were independently coded using the preliminary coding framework to refine and identify new codes. This initial coding stage was completed in Microsoft Word. The descriptions of the codes and sub‐codes were developed to capture the topic and intent of the related interview data. The remaining transcripts were then coded by the first author in NVivo (version 14) [29] using the coding framework. The consumer team member (J.M.) contributed to sense‐checking the codes and preliminary themes to ensure interpretation reflected lived experience. Coded data were exported from NVivo as an Excel spreadsheet, where data were organised into a matrix with rows (participants) and columns (codes and sub‐codes). Interpretive statements (themes) were constructed through an iterative process of reviewing codes, summary descriptions and data excerpts and comparing them across and within participants.

Visualisations were conducted in NVivo to explore the density and clustering of coding across the researcher and consumer groups, to identify trends and differences. Coding density and clustering are exploratory techniques for visualising patterns in data between groups. Illustrative quotes included a participant code and were designated as either ‘C’ for consumers or ‘R’ for researchers. One researcher participant had significant experience, both as a researcher and a consumer. This transcript was included in full for coding and analysis; quotes are denoted with an ‘R’ or ‘C’ in the ID, depending on the perspective from which the interviewee was speaking.

### 2.6 Reflexivity Statement

The qualitative analysis team consisted of two researchers and a consumer collaborator, who all identified as cis females. The researchers were healthcare professionals with PhDs and experience in qualitative research and methods for CCI in research. The consumer was an experienced health consumer with lived experience of stroke and other health conditions and had previously partnered in several research projects as a consumer co‐investigator. The interviews were conducted by the first author, who was newly appointed to the health service precinct and had no pre‐existing relationships with the interviewees or the research activities described in the interviews.

### 2.7 Ethical Considerations

Ethical approval was obtained from the Metro North Health Human Research Ethics Committee (approval number HREC/2024/MNHA/103152) and the University of Queensland (approval number: 2024/HE000829). No incentives were provided to consumer or researcher participants; consumers were offered paid parking if they completed the interview in person.

## 3 Results

### 3.1 Participant Characteristics

From the 54 participants who completed the online survey, 18 researchers and 10 consumers expressed interest in participating in an interview. All were invited to participate in an interview; however, one individual did not respond, resulting in 27 interviews conducted (health researchers, *n* = 17; consumers, *n* = 10). The characteristics of the participants are described in Table 1. The average interview length was 49 min, with a range of 30–109 min. Participants from both the researcher and consumer groups reported a wide range of CCI, from one–off contributions to consumer co‐led projects. Researchers reported a high level of CCI across their portfolios, with 71% stating that half or more of their research incorporated CCI. Participants reported positive experiences of CCI, with researchers (82%) and consumers (100%) describing their overall experience as good or excellent.

**Table 1:** Characteristics of interview participants regarding their experiences of CCI in research.

	*n* (%)
**Researchers (*n* = 17)**		
Lead employer	Health service	10 (59)
	University	7 (41)
Professional background	Allied Health	13 (76)
	Medical	1 (6)
	Sociology	2 (12)
	Technology	1 (6)
Age (years)	25–34 years	4 (24)
	35–44 years	6 (35)
	45–54 years	5 (29)
	55–64 years	2 (12)
Gender	Female	16 (94)
Research experience (years)	1–2 years	1 (6)
	3–5 years	6 (35)
	5–10 years	4 (24)
	> 10 years	6 (35)
How much of your research includes CCI	Little/Some	5 (29)
	Approximately half	2 (12)
	Most/All	10 (59)
Completed consumer training	Yes	9 (53)
Overall experience in CCI	Neutral	2 (12)
	Okay	1 (6)
	Good	5 (29)
	Excellent	9 (53)
**Consumers (*n* = 10)**		
Reported lived experience	Carer	2 (20)
	Physical Trauma	3 (30)
	Stroke (including aphasia)	2 (20)
	Disability	1 (10)
	Ageing	2 (20)
Age (years)	25–34 years	2 (20)
	45–54 years	1 (10)
	55–64 years	3 (30)
	65+ years	4 (40)
Gender	Female	2 (20)
Completed consumer training	Yes	7 (70)
Overall experience in CCI	Good	3 (30)
	Excellent	7 (70)

## 4 Themes

Themes were constructed as overarching statements that reflect the meaning and features of how participants described their experiences and understanding of the topics explored in the interviews.

### 4.1 Theme 1: Laying the Groundwork—Enabling Meaningful Consumer Partnering

This theme describes the foundational components that support high‐quality, effective and authentic CCI partnerships in health research. Participants across both cohorts emphasised the importance of early relationship building team culture, and a shared sense of purpose as critical to fostering genuine collaboration. For example, ‘…it's important to establish some rapport with people… there's something about your introductions and sharing your experiences, everybody equally, to get to know each other and also motivations’ (ID19.R).

Key enabling factors included role clarity, clear and effective communication, and responsiveness to consumers' needs. Specific adaptations, related to consumers' needs, to support their involvement were also highlighted, ‘…what's the best [communication medium] for the consumer rep? What do they prefer? What are their needs? What are their circumstantial needs?’ (ID2.C). Flexibility was a recurring topic of the interviews, which is captured in this theme. Flexibility was described as allowing extra time, flexible scheduling and adapting communication, which was central to accommodating circumstances and fostering participation from consumers.

In the workshops themselves, there's work that goes on from a traumatic brain injury point of view to enable those people with traumatic brain injury to be able to participate equally with the other stakeholders. So, what I mean there is if they've got cognitive impairments and communication impairments.

(ID7.R)

Flexibility was also discussed in the context of research methods, including adapting methods to encourage contributions based on consumers' abilities and preferences. This was accompanied by consumers describing valuable experiences in developing their research skills and capability through collaborative work with researchers.

Every step of those phases taking a moment, and pausing with him and going ok – ‘What could you see yourself doing in a phase like this? This is what is required. Do you think that your skills would be good in this phase? Or would you prefer to let us take the reins here a bit more?’

(ID24.R)

Personal sharing and lived experience were described as a compelling means of connection: ‘You can have a whole heap of people standing there talking about it, but when it's real, it comes across in an entirely different way because it's honest’ (ID9.C). Sharing lived experiences early in the partnership helped establish trust and set the tone for openness and mutual understanding. Researchers also described consumers' experiences as a powerful influence and advocacy platform that provides context and meaning to research, aiding in aligning people on research priorities and objectives.

I think that power of anecdote is a really important thing … to be able to say it in an emotional way. As well as using data and different forms of data. Cause if you just rely on the emotion, they say, ‘Oh, that's just one person.’ If you just rely on the statistics, they'll say, you know, ‘they're just dots on a page.’

(ID1.R)

Supportive teams that actively included consumers in leadership and decision‐making contributed to positive experiences for consumers. ‘It's important for me as a consumer rep to know that I have made a contribution that is valued …. That my input is valuable because it influences the outcome’. (ID2.C). Team dynamics mattered, balancing power and fostering collaborative rather than tokenistic involvement. Researchers felt that when roles were well‐defined and expectations were managed, it enabled effective partnerships.

It's about setting mutual expectations. And so for things like consumer advisory groups, we would start by spending time on developing a terms of reference. So that it's really clear to people. What we're asking them to do [research agenda]. And when and how and what their preferences are for that.

(ID8.R)

Researchers highly valued supportive processes that outsourced or streamlined the administrative component of consumer involvement, as shown by this researcher:

The process is kind of already really well‐established. So through [project] the admin has all the contact details and she's got all the paperwork set up so that any meetings I just say to her, ‘ these people attended this meeting’ and then she does all of the admin associated with that. The processes are all in place, so it happens seamlessly.

(ID15.R)

### 4.2 Theme 2: Opportunities: Organisational Barriers Influencing CCI

Despite executive leadership interest and policy support for CCI, participants described significant systemic and structural barriers that impeded authentic involvement. The administrative burden of onboarding and paying consumers was a recurring challenge.

Paying them [consumers] is really painful ‐ the process, so every time, I have to do a form for each of them, but they then have to sign and then they send it back to me. And then I sign it, and then I send it to my admin officer, and then the admin officer gets my director to sign it, and then it goes down to the business manager, and they'll eventually sign off on it, and then they might get paid a month or two later… It's administratively very burdensome.

(ID14.R)

This theme represents rigid, inaccessible systems, such as digital forms and financial processes, that were not consumer‐centric and often excluded those with limited digital literacy.

You're treated as second‐class citizens, a lot of us don't have good health literacy. So that's a lot of the problem. There's a huge gap between the consumer and those in the health system.

(ID6.C)

The limited flexibility of payment systems restricted the extent of consumer involvement. They described a consumer payment system designed for short, ad hoc, in‐person activities, which did not support deep involvement.

The process … with consumer payments is fine if you are doing ad hoc meetings or workshops. In terms of though, the consumer co‐leads, it was really prohibitive. It wasn't going to work, and it also, it really devalued the role that they were playing.

(ID10.R)

I think, firstly, having that payment enabler. So I have the choice if I want to do claim forms, I have the choice. If I wanted to have gift cards and I have the choice, if I want to invoice. I think that gives me autonomy and makes me feel valued.

(ID26.C)

Funding constraints were also described as a barrier. Without dedicated budgets or support structures, consumer involvement was sometimes added later in a project, reducing opportunities for co‐creation or early input. Organisational culture further shaped what was possible; when leadership did not prioritise or understand CCI, it was difficult to embed sustainable practices.

It's really hard, it's really, really hard to do research in a health service. So on one hand, we have all these amazing networks and processes to support consumer engagement that are quite well established, and it was very easy for us to tap into experienced consumers. But then it became really, really challenging to figure out how we were going to pay them.

(ID27.R)

This theme highlights the disconnect between policy‐level support for CCI and the practical realities of implementing consumer involvement in health research. Participants called for more streamlined, flexible systems that reduce administrative burdens and actively support diverse forms of involvement.

### 4.3 Theme 3: Navigating the Human Factor— Interpersonal Dynamics

Participants described CCI in research as deeply relational. Participants reflected on both the enabling and challenging aspects of interpersonal dynamics that impacted the quality of CCI. Building genuine relationships and networks was seen as a prerequisite for finding consumers and successful partnerships. Many participants noted that strong relationships—grounded in respect, empathy and active listening—were essential to collaboration. These dynamics often took time to develop and were shaped by the skills of facilitators and the broader team culture.

The choice of the people who run these projects is very important. [Researcher] was exceptionally compassionate, listened and very slow‐paced. You didn't feel pushed. You felt valued in everything that you contributed. You know, this is what the consumer needs is to feel heard.

(ID9.C)

It's not the same as building trust with a health professional partner. I didn't anticipate that it would require more time and energy and different conversations… All they're talking about is their personal life, and so I realised I had to be more vulnerable than I was being to build that trust… and actually be okay with some of our meetings not ticking off objectives of the project where we're building a relationship and having some debriefing conversations. And again, I didn't build any of this into the grant. And so that has been challenging.

(ID14.R)

The concept of the right consumer for the right role was a common and important topic. When consumers were well matched to projects, this contributed to more fulfilling and effective partnerships. As one consumer described:

You gotta get the right person. They've got to be enthusiastic. They've got to be vocal. They've got to be able to be sociable and make connections with the research team. They've gotta be a learner. And so many times, people come in, and they're a whinger. This isn't the person you really need on your research project. You need someone who's a believer. That's positive. That's willing to guide. You know, I had bad experiences as a patient, but you put that aside and you go on.

(ID22.C)

Both consumers and researchers recognised that timing was crucial for consumers to be ‘ready’ to engage in research through their lived experience:

Time since injury of the lived experience person is actually very important. There's a lot of information that some years ago I could not reveal, I'm OK with it now… So I guess I'm saying the progress of time is important from an injury. I guess it's distance, personal distance from that injury is important. So, whilst researchers want the data, I have to also be able to release it, be comfortable with releasing that data, on my personal experience to then apply it to the research.

(ID2.C)

Skilled facilitation was highlighted as a critical enabler, particularly in balancing relational work with the task‐oriented goals of research projects. Facilitators played a key role in managing group dynamics, supporting consumers to contribute, navigating conflict or discomfort when it arose, while keeping to time and project objectives.

It is very important that when consumers are brought in that whoever chairs the meeting makes certain that everybody has a voice. For example, it is so often that one or two people will take control of the situation, so that's quite important. … It's also quite important to have on‐hand resolution thinking. We have two different opinions and just work a way through where people can agree to resolve a difference in opinion.

(ID21.C)

The elements of psychological safety and emotional labour were also highlighted as under‐recognised aspects of CCI. Research projects may intersect with consumers' experiences of trauma or marginalisation due to their health condition or background. Trauma‐informed approaches, psychological support and reflective practices were identified as important; however, some researchers felt that they were insufficiently prepared.

I've done a lot of advocacy right, in health and been on panels and stuff, going trauma is the price of admission, right? That's how I get in the room as a consumer, because I've had incredibly traumatic experiences, and so has everyone else who is sitting there. Like they've had kids, they've had things go wrong, you wouldn't wish on your worst enemy. And in order to communicate that this may be a problem in the hospital, I have to say that out loud.

(ID11.C)

### 4.4 Theme 4: Bridging Methods—Codesign and Consumer Involvement

Participants, particularly researchers, discussed the lines between co‐design and CCI, identifying them as complementary yet distinct approaches that require different skill sets. Successful co‐design required a safe and inclusive space for effective collaboration and mutual learning. ‘It's actually quite a nuanced process, and you have to create a very safe space to actually share their ideas and motivate them to action the ideas that they put forward. So, it's kind of like a team‐building activity in the background to keep people through that project' (ID5.R).

While co‐design was widely valued, it required specific skills and structural support to succeed. Participants reflected on the challenges of translating co‐design methods into virtual formats, which required innovation and planning. CCI in research and co‐design was described as mutually reinforcing when done well, each strengthening the other. Co‐design practices provided a structure to consumer involvement, while meaningful CCI grounded co‐design efforts in lived experience and relevance. Consumer involvement deepened researchers' understanding of the real‐world nature of health issues, challenged assumptions and informed more relevant decisions.

In codesign, where you're trying to get an output as consumer advisor … you're part of the thinking group, and that needs some, you know, I have to think to do that. You're there to represent consumers, and you're there for the researcher to represent the concerns.

(ID13.C)

Researchers acknowledged the balance between organisational constraints and the integrity of the process, as tensions arose from the inability to implement co‐design findings within a complex and layered health service system. This can result in difficult conversations with consumer partners who might lack insider knowledge of the health service.

Consumers put a lot of importance on the work that they do and the involvement. And so there were things about the project that were difficult to implement as an outcome of it, and it got a little bit frustrating running a co‐design process. And having the decision makers in [Health service] not necessarily follow exactly what was coming out of the co‐design process and maybe taking things in a different direction and I think having the ability within a team just to be open and transparent about that, but also sit with the frustration that everyone's feeling.

(ID27.R)

### 4.5 Theme 5: Paving the Way Forward—Solutions and Integration

The ‘Paving the way forward’ theme captures participants' visions for strengthening and embedding CCI more meaningfully within health research practice. Practical, accessible training was a strong priority. Participants called for real‐world examples, role playing and mentorship from experienced practitioners in consumer involvement.

I think it's that practical stuff and getting together with people in the community of practice, but where it's practical. Like, what do you do if someone has a meltdown in your meeting? Just absolutely like the how to, the what, the expenses, the things that you had to consider … the real nuts and bolts.

(ID11.R)

Long‐term support and investment in ongoing, experience‐based learning and communities of practice were highly valued, rather than one–off training. Researchers reported that improving understanding of CCI across the health service would help shift the culture and recognition in research, ‘particularly for some of our younger clinicians, those fundamental skills haven't developed yet, so then you're not really thinking about consumers' (ID18.R).

Leadership and cultural change were identified as essential drivers. Organisational buy‐in and embedding CCI into planning and budgets were seen as key to long‐term sustainability. Where leadership explicitly supported CCI, there was greater alignment between policy and practice.

It is ensuring that team leaders, directors, executive directors are on board and facilitate that within our service line, where there are guidelines or whatever around the support of consumer payments that is coming out of the service line budget. Because then it starts to set the scene as something that is expected.

(ID10.R)

Inclusive structures and user‐friendly processes would reduce barriers and increase participation from underrepresented groups, alongside training that is developed collaboratively with consumers.

It's important that if the health sector runs training, they run it with people who are good in the consumer area and focus efforts on how you use consumers to inject more into the training.

(ID6.C)

### 4.6 Summary of Findings

**Figure 1:**
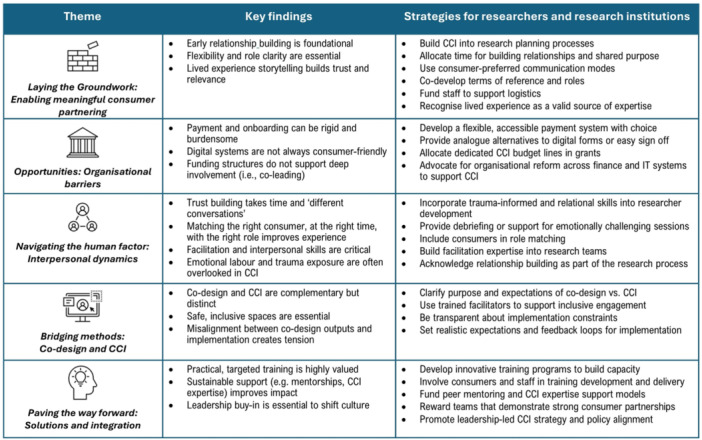
Summary of findings and strategies to promote meaningful consumer and community involvement (CCI) in health research.

To aid in translating findings into practice, Figure 1 presents summary findings and strategies for researchers, health services and research institutions to support meaningful CCI in research. These strategies were informed by insights shared by interview participants and approaches that would address the findings and themes identified in the data.

## 5 Discussion

This study explored the experiences of researchers and consumers regarding CCI at a large metropolitan hospital service precinct, identifying five interrelated themes that offer insight into what enables, challenges and sustains meaningful CCI. Findings reveal the complex, relational nature of CCI, where interpersonal, organisational and methodological factors intersect. Participants highlighted the foundational role of early relationship building, shared purpose and flexibility in establishing authentic partnerships. Meaningful CCI was facilitated by sharing personal experiences, clarity of roles and tailored communication strategies that respected individual capabilities and preferences. Consumers shared their motivations, passions and feelings, such as when they felt they belonged and were valued in their consumer role, highlighting the emotional aspects of CCI. Researchers emphasised the importance of building relationships but focused more on processes, planning and systemic issues, such as funding, organisational culture and administration. They acknowledged that existing systems may exclude groups that were important to work with, such as those with limited digital access or lower literacy. These perspectives highlight the importance of addressing both interpersonal and structural aspects of CCI. The nuances of CCI and co‐design were also discussed, highlighting the importance of facilitation skills and organisational support in successfully integrating consumer involvement into research methods. Participants proposed practical solutions for embedding CCI in more sustainable and inclusive ways, including capability building, more user‐friendly and efficient systems, leadership development, and structural support.

Our findings add to the literature on CCI in research by highlighting the context‐specific needs within complex hospital and academic research environments. Participants described inflexible payment systems and onerous administration as key barriers to authentic and diverse CCI, which is consistent with reports from similar healthcare settings [19, 30, 31]. Issues such as inaccessible forms, delayed payments and inefficient onboarding processes discouraged involvement and were burdensome for researchers and consumers. Participants advocated for a more inclusive and streamlined system to enhance accessibility, efficiency and transparency. These findings are supported by Ryan et al. (2024), who explored this topic in a similar Australian healthcare setting [19]. These issues point to the need to centre CCI practice and policy on equity and accessibility. While existing research has focused on CCI in specific contexts, such as focus groups [19], clinical trials [32] or cancer care [33], our study extends these findings and contributes a broader perspective by examining these issues across a large health service precinct that spans pre‐clinical, clinical and translational research. Our research findings also emphasised the importance of building workforce capability in CCI. This is supported by other studies that highlight workforce training as an essential component of CCI enablement [31, 32, 34]. The focus on personal experience and the value of lived experience support the view that CCI humanises research and helps shift power dynamics [35]. Trauma‐informed approaches in healthcare are well recognised, and there is a call to apply such approaches in the context of CCI [36, 37]. Our study captured the emotional labour of CCI and emphasised the need for researcher competence in trauma‐informed approaches. This supports the application of guidelines, such as the PPI trauma‐informed guidance for organisations and healthcare professionals released by Imperial College London and the National Institute for Health Research in 2024 [38]. These guidelines also acknowledge that professionals working with health consumers may be triggered by their own trauma or may hear concerning material disclosed through CCI activities [38]. This is highlighted in our findings by researchers who described feeling unprepared for the level of disclosure and vulnerability involved. Like consumers, researchers may also need opportunities for debriefing, reflection and support. Researchers should access their organisational support structures, such as supervisors, mentors or employee wellbeing services. Results from our study reinforce the importance of increasing awareness and competence in trauma‐informed practice for both consumers and researchers.

These findings have important implications, both within our local health service precinct and in other health service and research settings. Researchers with consumer expertise can play a pivotal role in modelling inclusive practices and advocating for cultural and attitudinal change within organisations. However, to avoid placing the burden of such change on individuals, organisations must actively foster supportive structures, shared responsibility and leadership in CCI. Administrative systems that streamline consumer onboarding, remuneration and payment processes, should be co‐designed with consumers and health researchers to ensure suitability and accessibility. Embedding CCI into research planning from the outset through funding support, including facilitation and training, will support more sustained and authentic involvement. This aligns with peak body recommendations, including the NHMRC and NIHR, to institutionalise CCI as a core element of responsible health research [9, 16]. The relational and emotional aspects of CCI should be recognised and addressed in project planning and training. This is underscored by the Capability Development Framework for building successful partnerships in healthcare, as described by Cox et al (2022) [13]. Where two of the three pillars of the Framework are assigned to ‘Personal Attributes’ and ‘Relationships and Communication’ [13]. These findings support the need for capability not only in CCI theory but also in facilitation, communication, interpersonal skills, and trauma‐informed approaches. Further research could explore the implementation of capability‐building programmes, including trauma‐informed practices, to determine the most effective ways to integrate such practices into CCI. In addition, these findings have highlighted perspectives on a lack of diversity among consumers involved in research. Work is needed to understand how inclusive CCI can be achieved with underrepresented groups, including those affected by complex trauma, indigenous populations and those with low literacy.

### 5.1 Strengths and Limitations

A strength of this study is the inclusion of viewpoints from both researchers and consumers, providing a comprehensive view of experiences. The use of the Framework method for qualitative analysis enabled a structured approach to interpreting the data. To reduce bias and promote more open responses, a researcher was appointed to conduct the interviews and analysis who was new to the organisation and therefore had no pre‐existing relationships with the participants. Additionally, the data processing methodology involved de‐identifying the transcripts before analysis with the broader team. Despite this, the implications of responder bias should be considered, given that the respondents were high adopters of CCI in health research. It would be of interest to explore the experiences of researchers and patients who do not engage in CCI to understand the barriers and enablers to their uptake of CCI practices; however, this was beyond the scope of this study. Although the study did not explore gendered issues, the uneven gender representation across the two samples (20% female among consumers and 94% female among researchers) may have influenced the perspectives shared. It should also be noted that the research was conducted within a particular health service precinct, which may limit the generalisability of the findings.

## 6 Conclusions

This study highlights that while CCI in health research is widely valued, its practice is often constrained by organisational systems, administrative burdens and a lack of sustained support. Meaningful CCI is enabled by early relationship building, role clarity, flexible processes, and strong interpersonal and trauma‐informed approaches. To move from policy to practice, health research institutions must invest in streamlined systems and structures that support long‐term, inclusive CCI. Researcher capability development should address not only the principles of CCI but also the interpersonal and facilitation skills essential for building successful and meaningful partnerships.

## Author Contributions

**Kimberley A. Baxter:** writing – original draft, study design, data collection, analysis, interpretation, writing – review and editing. **Jennifer Muller:** conceptualisation, study design, funding acquisition, analysis, interpretation and writing – review and editing. **David A. Copland** and **Nadine E. Foster:** conceptualisation, study design, funding acquisition, interpretation, writing – review and editing. **Emmah Doig**, **Jessica A. Schults**, **Karina R. Charles**, **Adrienne Young, Silvia Manzanero**, and **Tanya Smyth:** study design, funding acquisition, writing – review and editing. **Lisa Anemaat:** conceptualisation, study design, funding acquisition, writing – review and editing, analysis, interpretation, supervision, project administration, methodology.

## Ethics Statement

Ethical approval was obtained from the Metro North Health Human Research Ethics Committee (approval number HREC/2024/MNHA/103152) and the University of Queensland (approval number: 2024/HE000829). No incentives were provided to consumer or researcher participants; consumers were offered paid parking if they completed the interview in person.

## Conflicts of Interest

The authors declare no conflicts of interest.

## Supporting information

Supplementary_Materials_1.

Supplementary_Materials_2.

## Data Availability

Anonymised data that support findings are available on request from the corresponding author. Raw data are not publicly available to protect participant privacy.
