# Supplementary material for: Towards Meaningful Consumer and Community Involvement in Health Research: A Qualitative Study of Consumer and Researcher Experiences
Source: Health Expect. 2026 Mar 1;29(2):e70620. doi: 10.1111/hex.70620 (PMC12949962; doi:10.1111/hex.70620)
Supplement: Supplementary file 1 — Supplementary_Materials_1. [file HEX-29-e70620-s001.pdf]

Supplementary Materials 1.

GRIPP2 long form

Section and topic	Item	Reported on page No
Section 1: Abstract of paper		
1a: Aim	Report the aim of the study	Y, page 1
1b: Methods	Describe the methods used by which patients and the public were involved	Y, page 1
1c: Results	Report the impacts and outcomes of PPI in the study	Y, page 1
1d:Conclusions	Summarise the main conclusions of the study	Y, page 1
1e: Keywords	Include PPI, “patient and public involvement,” or alternative terms as keywords	Y, page 1
Section 2: Background to paper		
2a: Definition	Report the definition of PPI used in the study and how it links to comparable studies	Page 2
2b: Theoretical underpinnings	Report the theoretical rationale and any theoretical influences relating to PPI in the study	Page 2
2c: Concepts and theory development	Report any conceptual models or influences used in the study	N/A

Section 3: Aims of paper		
3: Aim	Report the aim of the study	Y, pg 2
Section 4: Methods of paper		
4a: Design	Provide a clear description of methods by which patients and the public were involved	pg 1 (PPI statement), pg 4-5
4b: People involved	Provide a description of patients, carers, and the public involved with the PPI activity in the study	Y, pg4, 5
4c: Stages of involvement	Report on how PPI is used at different stages of the study	Y, pg4, 5
4d: Level or nature of involvement	Report the level or nature of PPI used at various stages of the study	Y, pg 4,5
Section 5: Capture or measurement of PPI impact		
5a: Qualitative evidence of impact	If applicable, report the methods used to qualitatively explore the impact of PPI in the study	Y, pg 4,5
5b: Quantitative evidence of impact	If applicable, report the methods used to quantitatively measure or assess the impact of PPI	N/A
5c: Robustness of measure	If applicable, report the rigour of the method used to capture or measure the impact of PPI	Y, pg 4-5
Section 6: Economic assessment		
6: Economic assessment	If applicable, report the method used for an economic assessment of PPI	N/A
Section 7: Study results		
7a: Outcomes of PPI	Report the results of PPI in the study, including both positive and negative outcomes	Y, pg 6-11
7b: Impacts of PPI	Report the positive and negative impacts that PPI has had on the research, the individuals involved (including patients and researchers), and wider impacts	Y, pg 6-11
7c: Context of PPI	Report the influence of any contextual factors that enabled or hindered the process or impact of PPI	Y, pg 6-11
7d: Process of PPI	Report the influence of any process factors, that enabled or hindered the impact of PPI	Y, pg 6-11

7ei: Theory development	Report any conceptual or theoretical development in PPI that have emerged	N/A
7eii: Theory development	Report evaluation of theoretical models, if any	N/A
7f: Measurement	If applicable, report all aspects of instrument development and testing (eg, validity, reliability, feasibility, acceptability, responsiveness, interpretability, appropriateness, precision)	N/A
7g: Economic assessment	Report any information on the costs or benefit of PPI	N/A
Section 8: Discussion and conclusions		
8a: Outcomes	Comment on how PPI influenced the study overall. Describe positive and negative effects	Y, pg 11
8b: Impacts	Comment on the different impacts of PPI identified in this study and how they contribute to new knowledge	Y, pg 12-13
8c: Definition	Comment on the definition of PPI used (reported in the Background section) and whether or not you would suggest any changes	N/A
8d: Theoretical underpinnings	Comment on any way your study adds to the theoretical development of PPI	N/A
8e: Context	Comment on how context factors influenced PPI in the study	Y, pg 12
8f: Process	Comment on how process factors influenced PPI in the study	
8g: Measurement and capture of PPI impact	If applicable, comment on how well PPI impact was evaluated or measured in the study	Y, pg 13 (limitations)
8h: Economic assessment	If applicable, discuss any aspects of the economic cost or benefit of PPI, particularly any suggestions for future economic modelling.	N/A
8i: Reflections/critical perspective	Comment critically on the study, reflecting on the things that went well and those that did not, so that others can learn from this study	Y, pg 13 (strengths & limitations)

PPI=patient and public involvement
