# Supplementary material for: Towards Meaningful Consumer and Community Involvement in Health Research: A Qualitative Study of Consumer and Researcher Experiences
Source: Health Expect. 2026 Mar 1;29(2):e70620. doi: 10.1111/hex.70620 (PMC12949962; doi:10.1111/hex.70620)
Supplement: Supplementary file 2 — Supplementary_Materials_2. [file HEX-29-e70620-s002.docx]

**Supplementary Materials 2**

**Interview guide: RESEARCHERS**

***Consumer definition****: working as part of a research team (being involved in a research project/research-related activity in any way other than as a research participant).*

- Can you tell me about a research project you have been involved in with consumers?
  - Or tell me about a time when you have worked with a consumer in research
  - *What types of tasks/activities were consumers involved in?*
- Can you describe your experience working with consumers in research? / Can you describe your experience working with a research team? / How would you describe your experience?

***Prompts:***

- - *At what stages of the research project were consumers involved? (image support defining CCI research cycle stages provided to guide decision making)*
  - *Can you identify their level of involvement (according to the IAP2 – image support defining levels provided to guide decision-making)*
  - *What types of tasks/activities were consumers involved in?*
  - *How did you identify consumers (how were they invited)?*
  - *How many consumers contributed?*
  - *How were they paid for their involvement? Or were their contributions acknowledged in another way?*
- What do you think worked well in terms of consumer and community engagement?

***Prompts****:*

- - *Do you think you had enough knowledge to support consumers well?*
  - *Was there anything that helped you learn how to support the consumers involved?*
  - *What strategies for equalising power did you attempt, and what do you think worked the best?*
  - *Do you think the consumer was the right fit for the research?*
  - *How did you identify consumers for the research project?*
- When thinking about working with consumers/the research team throughout the project, what was the biggest challenge?
- Thinking about your experience, what would you suggest could help to improve/your experience of working with consumers/researchers on research projects?

**Prompts:**

- - *What do you think could help you and others in future? (e.g., resources, training, mentorship?)*
- Is there anything else you want to add that we didn’t ask?

**Interview guide: CONSUMERS**

***Consumer definition****: working as part of a research team (being involved in a research project/ related activity in any way other than as a research participant).*

- Can you tell me about a research project you have been involved in as a consumer?
  - *What types of tasks/activities were you involved in?*
- Can you describe your experience working with consumers in research? / Can you describe your experience working with a research team? / How would you describe your experience?

***Prompts:***

- - *At what stages of the research project were you involved? (image support defining CCI research cycle stages provided to guide decision making)*
  - *Can you identify the level of involvement you had (according to the IAP2 – image support defining levels provided to guide decision-making)*
  - *How were you invited to contribute?*
  - *How many consumers contributed?*
  - *How were you paid for your involvement? Or were your contributions acknowledged in another way?*
- What do you think worked well in terms of consumer and community engagement?

***Prompts****:*

- - *Did you feel supported? What made you feel supported/unsupported?*
  - *What did the team do that helped you feel part of the team and contribute to discussions and decisions?*
  - *Did you understand your role (in the research project)?*
  - *Did you feel your opinions were valued? Is there anything the team did that made you feel like your contributions were considered and valued?*
  - *Do you think you were the right consumer for this role?*
  - *Was there anything that helped or supported you?*
- When thinking about working with consumers/the research team throughout the project, what was the biggest challenge?

**Prompts**:

- - *Did you feel supported? What made you feel supported/unsupported?*
  - *Were there things that made it difficult for you to contribute?*
  - *Were there things that made you feel less like your contributions were important to the research project?*
  - *Was there anything that made it difficult for you to be involved/to involve consumers to the extent you wanted/intended?*
- Thinking about your experience, what would you suggest could help to improve/your experience of working with consumers/researchers on research projects?

**Prompts:**

- - *What do you think could help you and others in future? (e.g., resources, training, mentorship?)*
- Is there anything else you want to add that we didn’t ask?
